# Axon diameters and conduction velocities in the macaque pyramidal tract

**DOI:** 10.1152/jn.00720.2013

**Published:** 2014-05-28

**Authors:** L. Firmin, P. Field, M. A. Maier, A. Kraskov, P. A. Kirkwood, K. Nakajima, R. N. Lemon, M. Glickstein

**Affiliations:** ^1^Sobell Department of Motor Neuroscience and Movement Disorders, Institute of Neurology, University College London, United Kingdom;; ^2^Research Department of Cell and Developmental Biology, University College London, United Kingdom;; ^3^FR3636 Centre National de la Recherche Scientifique/Université Paris Descartes and Université Paris Diderot, Sorbonne Paris Cité, France; and; ^4^Department of Physiology, Faculty of Medicine, Kinki University, Osaka, Japan

**Keywords:** macaque, corticospinal, axon, conduction velocity

## Abstract

Small axons far outnumber larger fibers in the corticospinal tract, but the function of these small axons remains poorly understood. This is because they are difficult to identify, and therefore their physiology remains obscure. To assess the extent of the mismatch between anatomic and physiological measures, we compared conduction time and velocity in a large number of macaque corticospinal neurons with the distribution of axon diameters at the level of the medullary pyramid, using both light and electron microscopy. At the electron microscopic level, a total of 4,172 axons were sampled from 2 adult male macaque monkeys. We confirmed that there were virtually no unmyelinated fibers in the pyramidal tract. About 14% of pyramidal tract axons had a diameter smaller than 0.50 μm (including myelin sheath), most of these remaining undetected using light microscopy, and 52% were smaller than 1 μm. In the electrophysiological study, we determined the distribution of antidromic latencies of pyramidal tract neurons, recorded in primary motor cortex, ventral premotor cortex, and supplementary motor area and identified by pyramidal tract stimulation (799 pyramidal tract neurons, 7 adult awake macaques) or orthodromically from corticospinal axons recorded at the mid-cervical spinal level (192 axons, 5 adult anesthetized macaques). The distribution of antidromic and orthodromic latencies of corticospinal neurons was strongly biased toward those with large, fast-conducting axons. Axons smaller than 3 μm and with a conduction velocity below 18 m/s were grossly underrepresented in our electrophysiological recordings, and those below 1 μm (6 m/s) were probably not represented at all. The identity, location, and function of the majority of corticospinal neurons with small, slowly conducting axons remains unknown.

the corticospinal tract (CST) of primates is the largest descending pathway from the brain to the spinal cord. In addition to its role in the control of movement, it is also involved in other functions, such as control of afferent input, autonomic function, and long-term plasticity (Lemon 2008). CST axons originate not only from classical motor cortex but from other widespread regions of the frontal and parietal lobes (Dum and Strick 1991, 2005; Murray and Coulter 1981; Nudo and Masterton 1990; Porter and Lemon 1993). In primates, terminals from the CST are found throughout the spinal grey matter and are known to influence dorsal horn, segmental, and propriospinal neurons as well as motoneurons (Alstermark et al. 1999; Armand et al. 1997; Kuypers 1981; Porter and Lemon 1993). The pattern of termination varies across different primate species (Kuypers 1981; Lemon and Griffiths 2005). In the Old World macaque, corticospinal projections from the M1 hand area terminate most heavily in the contralateral lamina VII (Morecraft et al. 2013). The second strongest projection is to the contralateral motor nuclei in the lower cervical segments (Armand et al. 1997; Morecraft et al. 2013). These direct cortico-motoneuronal connections (Bernhard et al. 1953; Rathelot and Strick 2006) are particularly strong for hand and digit muscles (Armand et al. 1997; Maier et al. 2002; Morecraft et al. 2013; de Noordhout et al. 1999; Porter and Lemon 1993). Complete lesions of the pyramidal tract (PT) produce characteristic deficits in the control of distal muscles (Lawrence and Kuypers 1968; see Lemon et al. 2012; Tower 1940).

Using the light microscope, Lassek (1941) counted 554,000 axons in each PT of the macaque, and in a study of 58 macaques, Russell and DeMyer (1961) found an estimated total of 435,627 axons in each tract. The largest axons (including the myelin sheath) have been reported to be up to 13 μm in diameter (Häggqvist 1937; Porter and Lemon, 1993), whereas the smallest were reported to have a diameter of around 1 μm. Subsequently, an electron microscope study revealed even more axons smaller than 1 μm (Ralston et al. 1987). It is likely that many of these fine axons were not detected in the earlier light microscopy investigations.

Thus the macaque CST contains fibers with more than a 100-fold range in axon diameters. It seems unlikely that fibers of such disparate size have the same function. Decades of investigation into the physiological properties of corticospinal neurons in awake, behaving monkeys, which began with Evarts (1965), have been for the most part dominated by recordings from large neurons with fast-conducting axons (Baker et al. 2001; Evarts 1965; Kraskov et al. 2009; Lemon et al. 1986; Vigneswaran et al. 2013). In humans, it is known that noninvasive electrical or magnetic stimulation of CST evokes motor effects that are almost entirely the result of the fastest-conducting components of the tract (di Lazzaro et al. 2008; Edgley et al. 1997).

As a first step toward probing the function of the many small and slowly conducting fibers in the corticospinal system, we gauged the extent to which electrophysiological recordings reflect the full size range of corticospinal neurons. We compared the distribution of axon diameters in the PT with measurements of antidromic and orthodromic latencies recorded from large numbers of corticospinal neurons identified by electrical stimulation. The results demonstrate that the great majority of corticospinal neurons probably escape detection by standard electrophysiological recording, and that our knowledge of corticospinal electrophysiology is based almost entirely on neurons with fast axons that make up only a tiny fraction of the total present in the corticospinal system.

## METHODS

All relevant experimental procedures were approved by the Local Ethical Procedures committee and performed in accordance with the United Kingdom Animals (Scientific Procedures) Act.

### Neuroanatomical Study

#### Perfusion and preparation of material.

Two purpose-bred adult monkeys (GM: male, *Macaca mulatta*, age 8 yr, body weight 8.3 kg; and CS28: male, *M. fascicularis*, 3 yr, 4.5 kg) were deeply anesthetized with an intraperitoneal injection of sodium barbiturate (75 mg/kg) and then perfused through the heart with 0.1 M phosphate-buffered saline (PBS), followed by fixative (1% paraformaldehyde plus 1% glutaraldehyde in PBS). The brain and cervical spinal cord were dissected into small blocks of tissue, including one from the medulla and including the PT. The blocks were left in the fixative for 24–48 h. Transverse sections 200 μm thick, cut orthogonal to the long axis of the PT, were then cut on a Vibratome and placed back in fixative overnight at 4°C. Selected sections of the PT at the level of the olive were stained for 1.5 h in 1% osmium tetroxide and dehydrated through ascending alcohols (epoxypropane, 50% epoxypropane-resin mixture) and left in resin overnight. The sections were then placed flat and polymerized in fresh resin in an oven for 24–48 h. Semi-thin 1-μm sections were stained with 1% toluidene blue for 20 s. For electron microscopy, 100-nm-thick sections of the selected area were collected on copper grids and stained with 1% uranyl acetate and 1% lead citrate.

#### Sampling and measuring: electron microscopy.

Sample areas to photograph were systematically selected by aligning the field of view at ×5,000 magnification with the upper left corner of the copper grid squares. In total, 244 samples were taken (129 in GM, 115 in CS28) at ×10,000 magnification. Each picture was overlaid with a square counting frame with an area measuring 120 μm^2^. Axons that lay tangential to the lower and left border of the counting frame or entirely outside the frame were excluded from the measurements. Inner axonal diameters, i.e., axon diameters excluding the myelin sheath, were measured along the minor axis to avoid overestimation of diameters in noncircular profiles (Leenen et al. 1985). Myelin thickness was measured separately at well-preserved sections and in the same plane as the axonal diameters wherever possible. Outer axonal diameter (including myelin sheath) was calculated as inner axonal diameter plus twice the myelin thickness. We used the results from the first animal (GM) to calculate the minimal number of diameter measurements necessary to establish a virtually equal theoretical diameter distribution for both animals and thus to establish the sample size needed from the second monkey (CS28). We bootstrapped (10,000 times) samples of different sizes (100, 200, 300, etc.) from the measurements in animal GM and compared these sampled distributions to the empirical distribution of the same animal. The sample size was considered to be sufficiently large if 95% of samples of a given sample size lay within the 95% confidence interval of the empirical distribution for every decile of the empirical distribution.

To test what a systematic underestimation of axon size due to noncircularity might have on the overall result, we also used an area-based measure based on the square root of the minor (*a*) and major (*b*) diameter of each axon. From this we calculated the diameter of the equivalent circular axon with the same area (given by √*ab*) and added twice the mean myelin thickness at the major and minor axis of that axon to give a corrected external diameter. The major axis was measured in every tenth axon in an ascending series of size, and the correction factor derived from this sample was then applied to whole distribution of minor axis measurements.

#### Sampling and measuring: light microscopy.

Light microscopy was performed only in monkey GM. Sample areas were selected using the field of view of the light microscope at ×1,000 magnification. A total of 110 photographs were taken at ×1,000 magnification. All pictures were overlaid with a 120-μm^2^ counting frame as described above. Only inner axonal diameters were measured, because the limitation imposed by image resolution did not allow us to establish myelin thickness with sufficient accuracy.

#### Estimation of the total number of PT axons.

The cross-sectional area of one PT (on the right side) was measured on a light microscopic photograph taken at the level of the olive at the midpoint of its rostrocaudal extent. Dorsally, the tract is bordered by the inferior olive. There is an abrupt transition from the sectioned PT fibers to the transverse fibers of the olive. To estimate the number of axons per square micrometer visible on the light microscopic samples, we divided the total number of all axons counted on all samples by the total area of all samples taken. The number of axons per square micrometer was applied to the cross-sectional area of one PT to estimate the total number of axons per PT visible under the light microscope. We followed the same approach to estimate the total number of axons per PT visible under the electron microscope.

### Electrophysiological Study

Data were recorded from 11 purpose-bred adult *M. mulatta* or *M. fascicularis* monkeys (4 males, weight range 4.2–10.1 kg; 7 females, 5.3–9.5 kg). Measurements were taken as part of other studies reported elsewhere (Kraskov et al. 2009, 2011; Maier et al. 2013; Townsend et al. 2006; Umilta et al. 2007; Vigneswaran et al. 2011).

#### Preparatory surgical procedures.

All surgical procedures were performed under deep general anesthesia with the use of aseptic procedures and were followed by full postoperative analgesic and antibiotic treatment (see Kraskov et al. 2009 for details). In brief, a headpiece was implanted for head restraint, and a single recording chamber was mounted to allow access to the inferior limb of the arcuate sulcus (for area F5 hand area recordings) and the middle third of the central sulcus (for M1 hand area recordings). In two monkeys, the chamber was mounted over the midline for recordings from the supplementary motor area (SMA). Two fine (240-μm shank diameter) tungsten stimulating electrodes were chronically implanted in the medullary PT. The final depth of each PT electrode was determined from the lowest threshold point (usually <20 μA) for activation of the short-latency antidromic volley recorded through the dura from the ipsilateral M1 cortex (see Fig. 3*A*).

#### Cortical recording of PT neurons.

Cortical recordings from pyramidal tract neurons (PTNs) were made extracellularly using a Thomas Recording 7- or 16-channel drive. The drive carried 3–5 glass-insulated platinum electrodes (shank diameter 80 μm, impedance 1–2 MΩ) with an interelectrode spacing of 300 μm. After preamplification (×20; Thomas Recording drive), the signals from each electrode were further amplified (typically ×500 or ×1,000) and bandpass filtered (0.3–10 kHz). Data were acquired with a 25-kHz sampling rate. A biphasic search stimulus (each phase 200 μS) of 250–400 μA was applied to the PT electrodes at a rate of ∼2 Hz. Neurons showing responses with an invariant latency (jitter <0.1 ms; see Fig. 3, *B* and *C*) to PT stimulation were identified as antidromic. The antidromic latency (ADL) of each PTN was measured from the beginning of the stimulus artifact to the onset of the action potential (vertical line in Fig. 3, *B* and *C*). In most cases the current threshold of the antidromic response for each PTN was also measured. Responses evoked from the PT were then confirmed to be antidromic by using the spontaneous spikes, discriminated online, to collide the antidromic spikes (Baker et al., 1999; see asterisks in Fig. 3, *B* and *C*). During these recordings, the monkeys were awake, sitting quietly and not performing any task. Recordings were carried out in regions of M1, F5, and SMA that were all identified by intracortical microstimulation (see Kraskov et al. 2009; Maier et al. 2002).

#### Recordings from corticospinal axons.

Orthodromic responses from single corticospinal axons were measured in terminal experiments carried out under deep general anesthesia. In these experiments monkeys were induced with ketamine (10 mg/kg im) and surgical preparation carried out using isoflurane (1.5–2.0% in oxygen). Recordings were carried out after intravenous administration of α-chloralose (50–80 mg/kg) and withdrawal of the inhalational anesthetic (Maier et al. 1998, 2002, 2013). PT stimulation was carried out either via previously implanted chronic electrodes (as above) or via a monopolar tungsten electrode, which was inserted just rostral to the obex and 0.5–1.5 mm from the midline. Corticospinal volleys in response to PT stimulation were recorded with a ball electrode from the surface of the dorsolateral funiculus (see Fig. 3*D*) at a rostral site (spinal level C4–C5) and a caudal site (C8). Intra-axonal recordings were made from the lateral funiculus at the C4–C6 level with glass microelectrodes filled with 3 M potassium acetate and having a DC resistance of 2–5 MΩ. Corticospinal axons were identified as responding to PT stimulation with an invariant latency (see Fig. 3, *E* and *F*). A search stimulus of 200 μA was used, and the threshold for responses was determined in most cases. Intracellular and surface recordings were digitized directly at 10 kHz. The membrane potential was monitored throughout each recording, but the criterion for continued recording was solely that of sufficiently well discriminable positive-going spikes. Recording from single axons lasted from less than 1 min up to 18 min. All responses were confirmed to follow trains of three shocks at 333 Hz. All orthodromic latency (ODL) measurements were made offline using Spike2 (V6) software (Cambridge Electronic Design, Cambridge, UK) and averaged from around 20 single responses. ODLs were measured from the beginning of the stimulus artifact to the peak of the action potential (Fig. 3, *E* and *F*).

#### Magnetic resonance imaging.

In three animals, we estimated the antidromic conduction distance by measuring the course of corticospinal axons from the site of stimulation in the medullary pyramid to the recording site in M1, guided by the anatomical findings of Morecraft et al. (2002, 2007). Measurements were made from structural magnetic resonance imaging (MRI) scans taken when the monkey was first used in experimental procedures (M40: 4.5 kg, age 2.7 yr; M41: 5.1 kg, 2.8 yr; M43: 4.8 kg, 3.3 yr). These scans were performed at 1.5 T with the monkeys under heavy sedation. Voxel size was 0.7 × 0.7 × 0.7 mm. Measurements derived from these scans were confirmed in a further adult macaque, from a more recent study, that was imaged at 3.0 T.

## RESULTS

### Neuroanatomical Study

#### Electron microscopy.

**Fig. 1.**
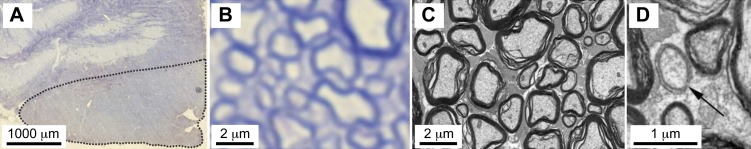
Neuroanatomical study. *A*: low-power light microscope image of monkey pyramidal tract (PT). The region measured as the area of the PT is outlined. *B*: high-power light microscope image of PT fibers. *C*: electron microscope image of PT fibers (case GM). Magnification is similar to that in *B*. The resolution is clearer than is possible with the light microscope. *D*: higher power electron microscope image. Note the single myelin wrapping (arrow). Although this configuration could represent an axon segment approaching a node, the frequency with which this type of profile was seen suggests that it probably represents a thinly myelinated axon.

A total of 4,172 axons were sampled in two macaque monkeys (GM: *N* = 2,355 axons; CS28: *N* = 1,817 axons). Ninety-nine percent of axons were myelinated (Fig. 1*C*). The median inner axonal diameter, i.e., the axonal diameter excluding the myelin sheath, was 0.68 μm (mean 0.91 μm, range 0.04–9.48 μm). The median axon diameter including the myelin sheath was 0.97 μm (mean 1.32 μm, range 0.10–12.73 μm). The few unmyelinated axons (*n* = 42, 1% of the sample) had a median diameter of 0.36 μm (mean 0.40 μm, range 0.13–1.17 μm). In addition to the small number of unmyelinated axons, there were several small axons with a single wrapping of myelin (Fig. 1*D*). These were classified as myelinated axons.

**Fig. 2.**
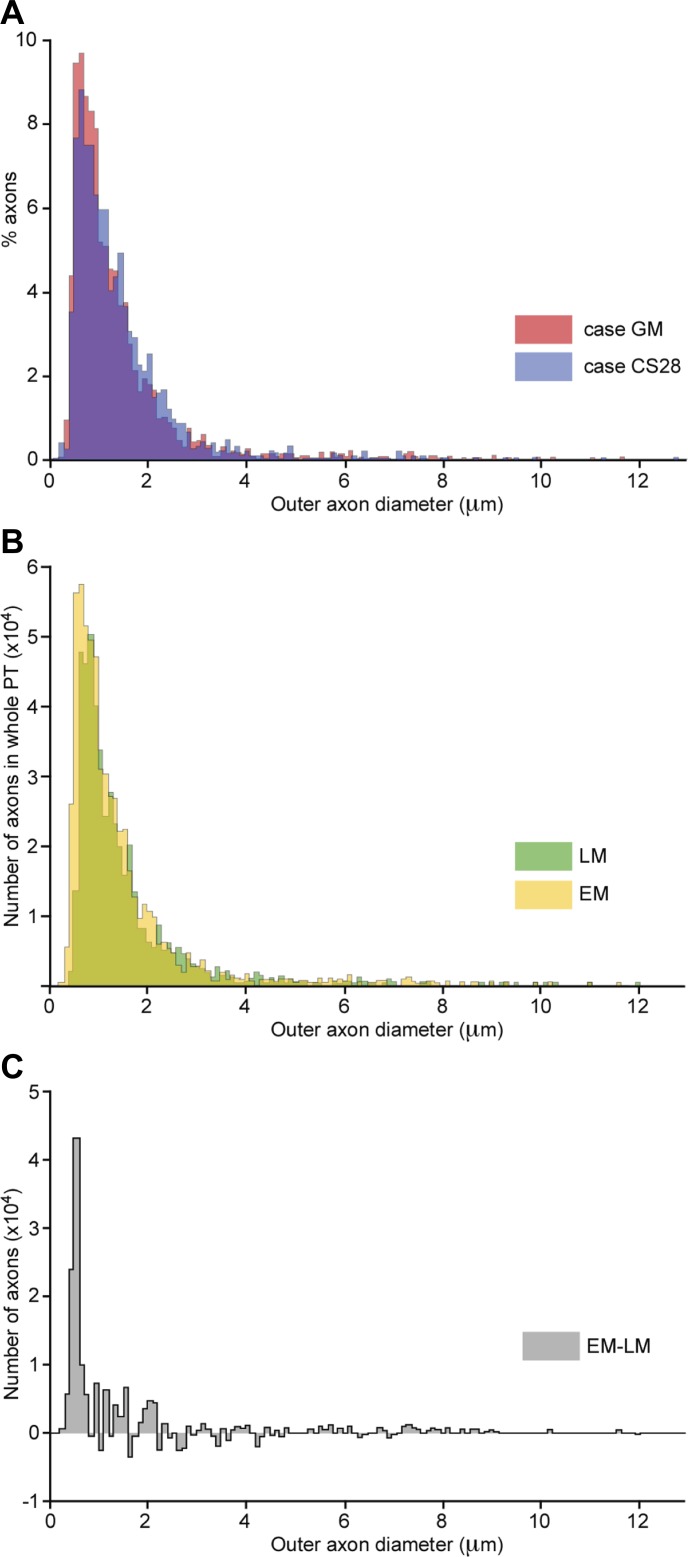
Distribution of axon diameters in the PT. *A*: distribution of outer axon diameters (axon plus myelin sheath) measured from axons in the PT in cases GM (2,355 axons, red) and CS28 (1,817 axons, blue), showing overlap of the 2 distributions (purple). Note the bias toward thin axons. *B*: comparison of distribution of outer axon diameter as measured in case GM using light microscopy (LM) vs. electron microscopy (EM). The distributions, derived from sampled areas, are scaled to represent the totals according to the total numbers of fibers estimated using each method. *C*: subtraction of the 2 main histograms in *B* shows that most of the difference between the 2 counts is in the finest axons (<0.6 μm).

The distribution of axon diameters was heavily biased toward small axons (Fig. 2*A*); 52% of axons were smaller than 1 μm, and 14% were smaller than 0.5 μm. Data from each of the two monkeys are given in Table 1, which shows that the results from the two cases were in good agreement.

**Table 1.** Axon diameters measured using electron microscopy

	Both Animals				CS28				GM			
Total no. of axons	4,172				1,817				2,355			
No. myelinated	4,130 (99%)				1,801 (99%)				2,329 (99%)			
No. <1 μm	2,155 (52%)				870 (48%)				1,285 (55%)			
No. <0.5 μm	565 (14%)				216 (12%)				349 (15%)			
Axon diameters	Med	Mean	Min	Max	Med	Mean	Min	Max	Med	Mean	Min	Max
Ø all inner	0.68	0.91	0.04	9.48	0.73	0.94	0.04	9.21	0.64	0.88	0.13	9.48
Ø myelinated inner	0.68	0.91	0.04	9.48	0.73	0.95	0.04	9.21	0.65	0.89	0.13	9.48
Ø unmyelinated	0.36	0.40	0.13	1.17	0.37	0.41	0.25	0.59	0.34	0.39	0.13	1.17
Ø all outer	0.97	1.32	0.10	12.73	1.03	1.37	0.10	12.73	0.91	1.28	0.13	11.53

Numbers of axons are given for both animals and for cases CS28 and GM, with percentages in parentheses. Median (Med), mean, minimum (Min), and maximum (Max) axon diameters are given in μm. Ø, diameter; inner diameter excludes and outer diameter includes myelin sheath.

The myelin-to-axon thickness ratio was variable (range 0.003–4.41), with a mean of 0.23, meaning that on average the myelin sheath amounted to about 32% of total diameter (cf. Bischoff and Thomas 1975; Bishop et al. 1971; Hildebrand and Hahn 1978; von Keyserlingk and Schramm 1984), and myelin thickness was strongly correlated with diameter over the entire range of axon size (Pearson correlation coefficient *r* = 0.83).

#### Light microscopy.

Light microscopy was performed only on sections from monkey GM. We sampled 1,658 axons with a median inner diameter (excluding the myelin) of 0.78 μm (mean 0.98 μm, range 0.25–9.01 μm; Fig. 1*B*). Myelin thickness could not be reliably measured using light microscopy (see methods), so we estimated the total axon diameter using the myelin-to-axon ratio of 0.23 that was found with electron microscopy (i.e., multiplying the inner diameter by 1.46). The estimated median axon diameter including the myelin was 1.03 μm (mean 1.43 μm, range 0.37–13.07). In this study, no unmyelinated axons could be identified using the light microscope.

#### Number of axons in the PT.

Based on light microscopy of the PT of monkey GM, we measured a PT area of 3.782 mm^2^ for one tract (Fig. 1*A*). The total area in our light microscopic study occupied by all the axons sampled in GM was 0.013 mm^2^; accordingly, the estimate of the total number of axons per PT (to the nearest 1,000 axons) was 482,000 axons. For the number of axons counted using the electron microscope, the total sampled area was 0.015 mm^2^, and the resulting estimate of the total number of axons was 594,000.

#### Comparison between electron and light microscopy.

Using the electron microscope, we measured 63 myelinated and 26 unmyelinated axons smaller than 0.25 μm. Although a few 0.25-μm axons (corresponding to a myelinated diameter of 0.36 μm) were detected with high-resolution light microscopy, systematic counts with the EM make it clear that most of the smallest axons in monkey GM would have remained undetected using light microscopy. Based on a comparison of light and electron microscopic counts, we estimate that the total number of axons detected was 19% lower using the light microscope. Most of those undetected would have had a myelinated diameter <0.6 μm (Fig. 2, *B* and *C*).

### Electrophysiological Study

#### Antidromic responses in PTNs.

##### LATENCIES.

**Fig. 3.**
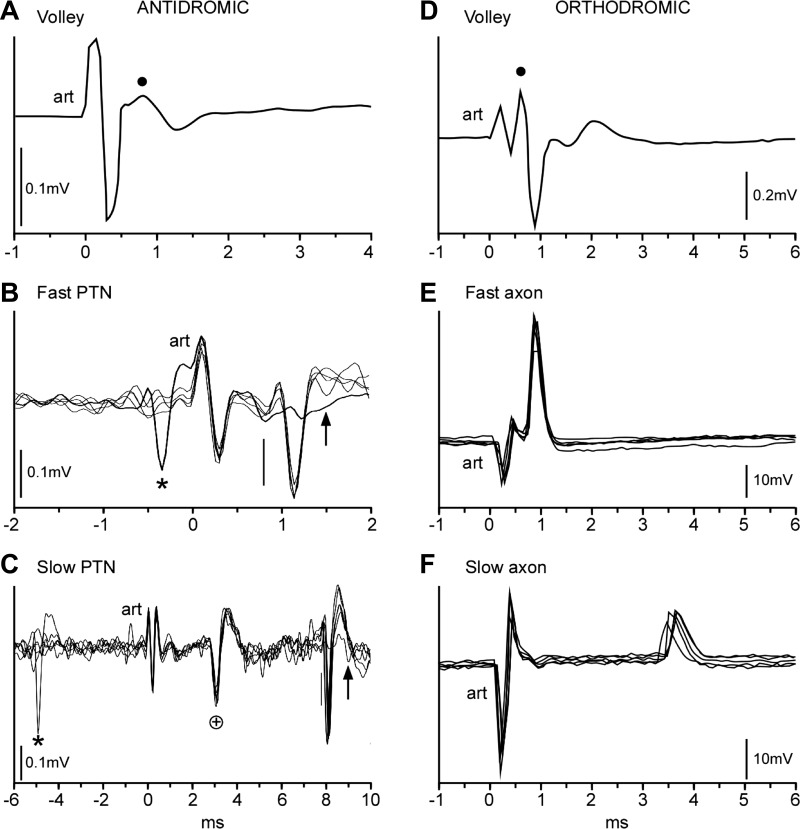
Neurophysiological study. *A–C*: antidromic responses of PT neurons recorded in motor and premotor cortex. In all records, positivity is up. *A*: antidromic volley recorded transdurally from M1 cortex during implantation of a stimulating electrode in the ipsilateral PT (case M41): average response (*n* = 100 sweeps, intensity 200 μA). art, Stimulus artifact. Dot marks positive peak of response. *B*: example of an extracellular recording of a PT neuron (PTN) in M1 with a short antidromic latency (0.8 ms, measured from stimulus onset at *time 0* to vertical bar). Four superimposed sweeps show that the response had an invariant latency, and its antidromic nature was confirmed by collision with a spontaneous spike (*); note absence of the antidromic response in this sweep (arrow). *C*: example of a PTN with a long antidromic latency (8 ms) recorded in area F5. Five superimposed sweeps show that the response had an invariant latency, and its antidromic nature was confirmed by collision with a spontaneous spike (*); note absence of the antidromic response in this sweep (arrow). Another PTN with a shorter antidromic latency (+) at 2.6 ms was also present in the record; this spike was not collided. *D* and *E*: orthodromic responses of corticospinal axons recorded from the mid-cervical cord. *D*: averaged orthodromic volley (*n* = 44 sweeps, intensity 200 μA) recorded from the dorsal surface of the spinal cord. Dot marks positive peak of response. *E*: 5 superimposed records from an axon with a short latency response (0.9 ms). *F*: 5 superimposed records from an axon with a long latency response (3.6 ms).

Antidromic latencies of PTNs were recorded in seven awake macaques. We sampled 589 PTNs in M1, 147 PTNs in ventral premotor area F5, and 63 in SMA. Examples of neurons with a short ADL (from an M1 PTN) and long ADL (from an area F5 PTN) are shown in Fig. 3, *B* and *C*, respectively. Antidromic thresholds ranged from 10 to 360 μA, with a median of 120 μA. In M1, the median antidromic latency (ADL) across all animals was 1.1 ms (mean 1.47 ms, range 0.5–8.7 ms). In area F5, the median ADL was 2.3 ms (mean 2.47 ms, range 0.7–8.7 ms). For the SMA, the median ADL was 1.7 ms (mean 2.11 ms, range 0.8–6 ms). Results from each of the monkeys are shown in Table 2.

**Table 2.** Electrophysiological latencies

Case	Sex	Side	PTNs/Axons	Min	Max	Median
*Antidromic latencies PT–M1*						
M38	F	L	127	0.6	3.7	1.0
M39	F	L/R	76/121	0.6	4.4	1.2
M40	M	R	14	0.9	1.8	1.3
M41	M	L/R	66/50	0.7	4.4	1.1
M43	F	R	50	0.5	5.7	1.1
M32	M	L/R	40/19	0.7	8.7	1.2
M34	F	R	26	0.7	2.4	1.1
*Antidromic latencies PT–F5*						
M39	F	L/R	2/13	0.9	2.8	1.8
M40	M	R	10	1.1	3.9	1.9
M41	M	L/R	38	0.7	4.4	1.8
M43	F	R	84	1.1	8.7	2.6
*Antidromic latencies PT–SMA*						
M32	F	L/R	13/14	0.8	6.0	1.8
M34	F	R	36	1.1	5.1	1.7
*Orthodromic latencies PT–C4-C6*						
CS15	M	*	26	0.8	1.7	0.9
CS22	F	*	73	0.6	2.3	0.8
CS23	F	*	24	0.7	1.3	0.8
CS28	M	*	51	0.8	3.6	1.0
M40	M	*	18	0.8	2.1	1.1

Min, Max, and median latencies are given in ms for the indicated no. of pyramidal tract neurons (PTNs) and axons.

F, female; M, male; L, left; R, right.

* All axon recordings were made from mid-cervical segments on the right side.

##### LATENCY DISTRIBUTION.

**Fig. 4.**
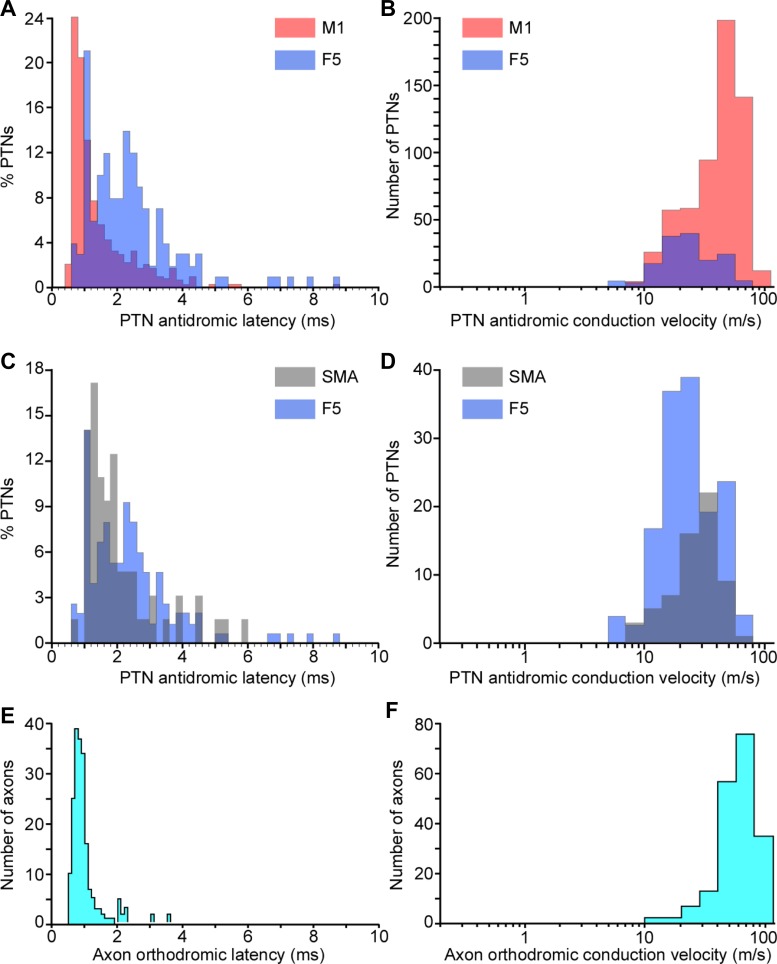
Distributions of response latencies and conduction velocities for corticospinal neurons. *A*: distribution of antidromic latencies of PTNs recorded in M1 (*n* = 589; red) and F5 (*n* = 147; blue) in 7 and 4 awake macaque monkeys, respectively. Overlap of these 2 distributions is shown (purple). Note the bias toward short-latency effects and the presence of a faster population of PTNs in M1, which is lacking in area F5. *B*: distribution of conduction velocities for the same data based on a conduction distance of 47 mm (see text). *C*: distribution of antidromic latencies of PTNs recorded in SMA (*n* = 63, gray) and F5 (*n* = 147, blue, as above) in 2 and 4 awake macaque monkeys, respectively. Again, note the bias toward short-latency effects. *D*: distribution of conduction velocities of the data shown in *C*. *E*: distribution of orthodromic latencies of corticospinal axons recorded at C4–C6 (*n* = 192); again, note bias toward short-latency effects. *F*: distribution of conduction velocities for the same data, based on a conduction distance of 40 mm (see text). Note log scale for *B*, *D*, and *F*.

The distribution of ADLs was strongly biased toward short latencies (Fig. 4*A*). The shortest ADL was 0.6 ms and the longest 8.7 ms. ADL distributions recorded in M1 (Fig. 4*A*, red) differed significantly from distributions recorded in area F5 (Fig. 4*A*, blue). Similarly, the distribution of ADLs in SMA (Fig. 4*C*, gray) differed from that in F5 (Fig. 4*C*, blue) and that in M1 (cf. Fig. 4*A*). Kolmogorov-Smirnov tests confirmed significant differences between these three ADL distributions (M1 vs. F5: *D* = 0.44, *P* < 0.001; M1 vs. SMA: *D* = 0.49, *P* < 0.001; SMA vs. F5: D = 0.27, *P* < 0.002). In one animal (M39), we were able to compare the distribution of ADLs for PTNs recorded in M1 of the left hemisphere (76 PTNs) with that of the right (121 PTNs; see Table 2). No significant difference between the two hemispheres was found (Kolmogorov-Smirnov test, Monte Carlo-based bootstrap, *P* > 0.05).

##### CONDUCTION VELOCITIES.

The distance from the PT stimulation site to the recording site in M1, based on MRI measurement in three of the monkeys, was estimated to be 47 mm (estimates in the 3 monkeys were 44, 47, and 47 mm). This value is similar to the estimate of 46–48 mm made by Humphrey and Corrie (1978) from their “stereotaxic reference atlas.” Figure 4*B* shows the distribution of conduction velocities calculated for this distance from the ADLs in Fig. 4*A*, assuming a utilization time of 0.1 ms. Note the log scale. For all M1 PTNs, the median conduction velocity was 47 m/s (mean 46 m/s, range 5–94 m/s). For all F5 PTNS, the median was 21 m/s (mean 26 m/s, range 5–78 m/s; Fig. 4, *B* and *D*). For all SMA PTNs, median was 29 m/s (mean 29 m/s, range 8–67 m/s; Fig. 4*D*).

#### Orthodromic recordings from corticospinal axons.

Recordings were made from a total of 192 axons in the cervical spinal cord of five anesthetized macaques; one of these animals had also been used for the awake study reported above. Examples are shown in Fig. 3, *E* and *F*. Orthodromic thresholds ranged from 10 to 430 μA, with a median of 95 μA.

##### LATENCIES.

The shortest latency was 0.6 ms and the longest 3.6 ms, and the ODL distribution for all axons was even more strongly biased toward short latencies than was the ADL distribution (Fig. 4*E*). Very few long-latency responses were recorded. Separate results from each of the monkeys are shown in Table 2.

##### CONDUCTION VELOCITIES.

A conduction distance of 40 mm was assumed (Humphrey and Corrie 1978). Conduction velocities calculated for this distance are shown in Fig. 4*F* on a log scale; conduction times were corrected by subtracting 0.1 ms for utilization time plus a further allowance of 0.1 ms for the rise time of the intra-axonally recorded action potential. The median value was 57 m/s (mean 58 m/s, range 12–100 m/s).

Gross potential volleys in the PT were recorded at two recording sites, at spinal levels C5 and C8, in three animals. From the distance between these two recording sites and the difference in ODL of the fastest component of the volley at each site, we calculated the conduction velocity of this component to be 70 m/s [CS15: 63 m/s (29 mm in 0.46 ms); CS28: 74 m/s (14 mm in 0.19 ms); M40: 73 m/s (27 mm in 0.37 ms)].

## DISCUSSION

We measured the distribution of axon diameters in the macaque PT using light and electron microscopy and compared the results with electrophysiological recordings of antidromic and orthodromic latencies in response to PT stimulation. The results indicate that neurons having small-diameter axons (around 1 μm or less), which account for more than half of the axons in the PT, were not sampled using the standard electrophysiological techniques that were employed in this and similar studies. Although the antidromic identification of a neuron as a PTN cannot alone provide insight into its function, it is a necessary first step if we are to understand the role of different classes of cortical projection neurons (Evarts 1965; Fetz and Cheney 1980; Lemon et al. 1986; Quallo et al. 2012; Vigneswaran et al. 2013). This is particularly true of the neurons making up the most slowly conducting component of the CST.

### Number of Axons in the Medullary PT

Based on our counts of samples under the electron microscope, we estimated there to be a total of 594,000 axons in the PT of monkey GM. This number is slightly greater than previous estimates using the light microscope (Lassek 1941 and Russell and DeMyer 1961: 554,000 and 453,000, respectively), as well as our own estimate based on light microscopic counts (482,000). Our electron microscopy-based estimates were about 19% higher than those based on light microscopy. Although axons as small as ∼0.25 μm might occasionally be identified with the light microscope, those with a diameter smaller than ∼0.5 μm are probably routinely missed under the light microscope.

### Size Distribution in Pyramidal Tract Axons

The distribution of axon diameters found in the two examined animals was virtually identical (Table 2) and confirms previous reports that this distribution is heavily biased toward small diameters. Most axons were very thin. We found very few unmyelinated axons in the PT (around 1% of axons), in agreement with Ralston et al. (1987). In contrast, there were many thinly myelinated axons, partly with a single or incomplete myelin coating. Using the data measured at the electron microscope level, we estimate that around 52% of fibers are smaller than 1 μm (axon plus myelin sheath) and only 0.06% are larger than 3 μm. Therefore, compared with earlier studies, which did not employ electron microscopy to quantify the distribution of corticospinal axons (Häggqvist 1937; Russell and DeMyer 1961), our study shows that there are many more fine fibers than previously shown, further underlining the mismatch between neuroanatomical and electrophysiological measures. The distribution of fiber diameters measured in the electron microscope in our study is remarkably similar to that measured in the electron microscope for human PT axons by von Keyserlingk and Schramm (1984), except that the bias toward very small axons was even stronger in our study.

### Conduction Velocity-Axon Diameter Relationship in the PT

**Fig. 5.**
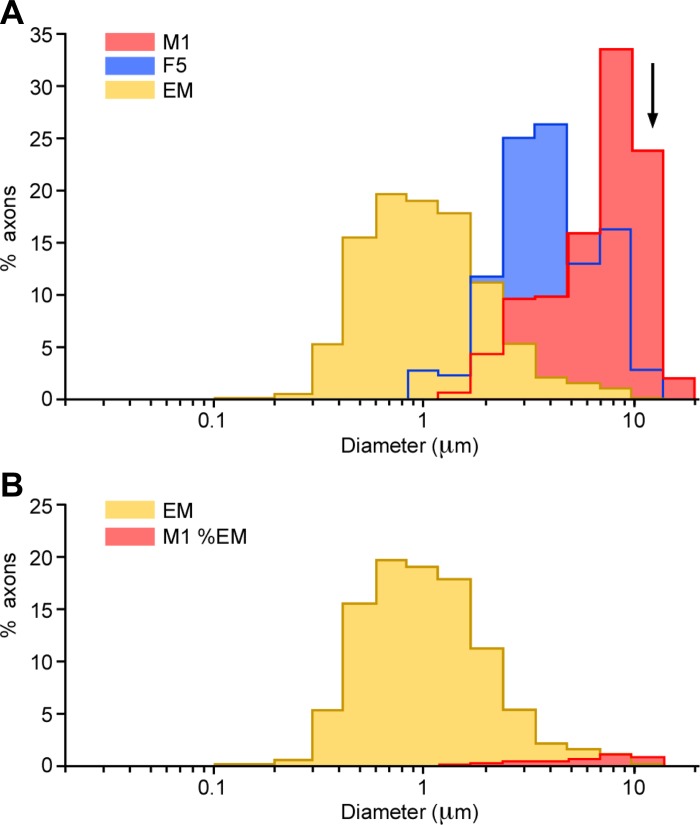
Comparison between the axons of corticospinal neurons sampled electrophysiologically with the PT axons revealed in the EM. Distributions of outer axon diameters measured in the EM and of those estimated from the conduction velocity measurements were converted to axon diameter using a Hursh factor of 6. *A*: distributions plotted as percentages of their own populations. M1 and F5 groups are plotted separately. EM population data are pooled from cases GM and CS28. Arrow indicates the axon diameter (11.7 μm) corresponding to the mean conduction velocity (70 m/s) derived from dorsolateral funiculus volleys evoked by PT stimulation. *B*: with the assumption that the M1 antidromic group is a subgroup of a total population represented by the EM measurements, the scale of the M1 histogram has been reduced so that it fits within the envelope of the EM histogram. To do this, the scale was reduced by a factor of 31, indicating that electrophysiologically identified M1 corticospinal neurons are likely to constitute ∼3% of the total. Note the log scale for axon diameter.

Using the estimates of conduction distance for the antidromic recordings, we can calculate the conduction velocity of the sampled axons (Fig. 4, *B* and *D*). The range was from 5 to 94 m/s, with a strong bias toward fast-conducting axons, particularly in the M1 recordings (Fig. 4*B*). The “Hursh factor” relates the axon diameter to the conduction velocity (Hursh 1939) and, for large axons in peripheral nerves and in the central nervous system, has a value of around 6 (Swadlow and Waxman 1975). Figure 5*A* plots, on a log scale, the distribution of estimated outer axon diameters (axon plus myelin sheath), using a Hursh factor of 6, that would have produced the spectrum of conduction velocities found in our antidromic recordings. The overlap between the electron microscope measurements (yellow) and those estimated from the electrophysiological samples is entirely limited to the larger diameters, and this is particularly true of the M1 sample (red). Compound volley measurements from the CST, made with surface electrodes, are even less representative of the underlying fiber diameter distribution, as is apparent in the recordings of Maier et al. (2013). The mean value for the conduction velocity of the surface volley in the present data (70 m/s) is included in Fig. 5*A* (arrow) to emphasize that fact.

Figure 5*A* has been plotted on a scale that normalizes the numbers of neurons in the anatomical and physiological data sets. In Fig. 5*B*, we have replotted the data, assuming that the M1 data must be included within the total envelope of the EM measurements taken from the PT. To do this we had to reduce the total size of the M1 sample by a factor of 31. After this correction, the recorded M1 population amounts to around 3% of the total corticospinal output measured from the PT, and this reflects the true extent of the oversampling of fast-conducting neurons in our recordings. This sample represents a total of around 18,000 axons (3% of 594,000), which is still a sizeable population. The proportion of large fibers is probably higher for the corticospinal projection from M1 (estimated to be about 50% of the total frontal lobe projection; Dum and Strick 1991).

The longest antidromic latency measured in our sample (8.7 ms) would correspond to a neuron with an axon conduction velocity of around 5 m/s and a diameter of 0.8 μm. Thus neurons with axons smaller than this were not detected electrophysiologically in M1, F5, or SMA. In particular, records from neurons with axons having diameters smaller than 0.8 μm and with conduction velocities less than 5 m/s (Waxman and Swadlow 1977; Waxman and Bennett 1972) are entirely missing in our recordings, while those with axons having diameters of 0.8–2.5 μm (5–15 m/s) are greatly underrepresented. Tanji et al. (1978) and Fromm and Evarts (1981) reported somewhat higher proportions of fibers in ADL range 2–4 ms (around 15–30 m/s, 2.5–5 μm) than the present study, but the overall range of conduction velocities was similar, so the estimate of the proportion sampled electrophysiologically from the total shown by electron microscopy would be similar to that using our data.

In compiling Fig. 5, we have assumed a Hursh factor of 6. It could be that higher values might be applicable to the largest axons, whereas it might be substantially lower for very fine axons (Boyd and Davey 1968; Waxman and Swadlow 1977). If, for example, we used a Hursh factor of either 8.48 or 4.24 (which covers the entire range in the literature), then this would correspond to moving the M1 histograms in Fig. 5 one bin to the left or right, respectively, before fitting. We calculate that this would change the overlap of the M1 histogram to roughly 5% (Hursh = 8.48) or 0.4% (Hursh = 4.24), respectively, of the electron microscope total. Applying the lower value would remove almost all of the overlap between the two data sets.

Our estimate of axon diameter at the electron microscope level was based on measurement of the minor axes of profiles that were, in general, irregular and crenellated (see methods and Fig. 1*C*). Most previously published electron microscope studies of central pathways show similarly irregular profiles (e.g., Bishop et al. 1971; Leenen et al. 1985; Waxman and Swadlow 1976), and in many of these studies the minor axis has been the measurement of choice. For example, Leenen et al. (1985) advocated the measurement of the minor axis, not only to avoid errors due to those axons that might have been cut obliquely but also to avoid errors introduced by elliptical and other irregularly shaped profiles. Because measurement of the minor axis might underestimate axon diameters, for a randomly selected sample of 10% of the axons, we also derived an area-based measure, based on the square root of the minor times the major axis measurement, and then added twice the average myelin thickness at these axes (see methods). The correction factor calculated for each axon (corrected external diameter divided by original external diameter based on the minor axis) had a mean value of 1.32. When applied to the whole population data, this gave a median corrected outer axon diameter of 1.28 μm (mean 1.74 μm, range 0.13–16.80 μm), compared with 0.97 μm (mean 1.32 μm, range 0.10–12.73 μm).

The estimated proportion of fibers with a corrected diameter <1.0 μm in this sample was 37%, compared with 52% in the original data. If the correction factor was applied to the entire population of axons, it would have shifted the range of axon sizes to the right by less than one bin in Fig. 5, and the scaling of the physiological population to fit the electron microscope histogram in Fig. 5*B* would be unaltered.

In some studies, the cross-sectional area of the axon has been measured (e.g., von Keyserlingk et al. 1985) and the diameter of a circle having an equivalent area taken as the axon's diameter. However, it must be recognized that a potential drawback of area-based measures is that the “true” shape of PT axons in vivo is unknown.

### Sampling Bias in Electrophysiological Recordings

Our comparison demonstrates the strong bias in recording toward large neurons. This bias is actually greater in our spinal intra-axonal recordings than in the cortical cell recordings (compare Fig. 4, *B* and *F*), probably because of the difficulty of intra-axonal recording from the thinnest, slowest axons. The distribution of conduction velocities in the present study is almost identical to that of the population of PTNs recorded by Edgley et al. (1997) using the same technique. The bias might also reflect the fact that, at least in the rat, the thinnest PT axons do not seem to penetrate far down the spinal cord but terminate more rostrally (Leenen et al. 1985).

Neurons with fast-conducting axons generally have larger cell bodies than those with slow axons (Deschênes et al. 1979; Naito et al. 1969; Sakai and Woody 1988; Sloper and Powell 1979; Tomasi et al. 2012), although the precise relationship between cell body and axon size is far from established. However, it is plausible that PTNs with thin axons may have small cell bodies and so be difficult to isolate in cortical recordings. Indeed, for electrophysiological studies, there is a well-known sampling bias toward recording from neurons with large somata (Humphrey and Corrie 1978; Towe and Harding 1970), and generally, corticospinal neurons with axons conducting slower than 5 m/s are absent from published data [Humphrey and Corrie 1978 (monkey); Mediratta and Nicol 1983 (rat)]. Of course, in the rat, the fastest axons conduct at only about 19 m/s, and over half of the PT axons are reported to be unmyelinated (Leenen et al. 1985). The recording bias is probably worse with metal microelectrodes than with glass micropipettes, but even when high-impedance glass micropipettes were used by Macpherson et al. (1982) for recordings in macaque M1 and SMA, only a few PTNs conducting at <5 m/s were reported.

All our cortical recordings were carried out in awake monkeys, avoiding any effects of anesthetics on axon conduction or antidromic invasion and allowing study of spontaneous activity in all PTNs recorded, which in turn enabled us to use the collision test to confirm their antidromic identification. It is possible that neurons giving rise to the smallest axons were not active in the experimental conditions we used or that they cannot be antidromically invaded (Lipski 1981).

### Did the Stimuli Used Activate the Finest PT Axons?

It is well established that thresholds are higher for fine vs. thick axons, so we have to consider the possibility that the finest PT axons were not activated by the stimuli used in our study. At least three factors are worthy of consideration. First, the PT stimulating electrode configuration involved two sharp electrodes placed around 5 mm apart along the long axis of the tract, which should be optimal for activating PT fibers. Second, the duration of the stimulus pulse (200 μS) is in the range of published chronaxie measurements for fine intracortical or intraspinal fibers emanating from cortical pyramidal neurons (Nowak and Bullier 1998; Shinoda et al. 1976). Finally, the current intensity used was clearly adequate for activation of some slow fibers. The threshold for antidromic responses varied widely (10–360 μA); if the finer fibers in the PT had systematically higher current thresholds than the thick ones, one can predict that there ought to be a positive correlation between an axon's current threshold and its antidromic or orthodromic latency. In fact, we found either no correlation (for orthodromic responses) or a very weak one (*r* = 0.22, for antidromic responses). Some of the slowest antidromic responses we recorded (>4 ms) had relatively low thresholds (<100 μA). We hypothesize that proximity to the stimulating electrode is a more important factor in determining threshold than axon size. It is likely that some of the finest axons were located close to the tips of the stimulating electrodes, whereas others might be up to ∼1 mm away. If proximity to the tip of the stimulating electrode were a major factor affecting excitation threshold, this would explain the lack of a clear threshold-latency correlation. Future studies may be able to exploit optogenetic methods for exciting corticospinal fibers of different sizes (Petreanu et al. 2007).

### Fast- and Slow-Conducting PT Neurons

The fact that PTNs vary in their shape, size, dendritic organization, and electrophysiological features (Deschênes et al. 1979; Spain et al. 1991; Takahashi 1965; Vigneswaran et al. 2011) is evidence against a single function for the CST (Kuypers 1981; Lemon 2008). Early investigations clearly distinguished between functional features related to “fast” vs. “slow” PTNs (Calvin and Sypert 1976; Evarts 1965; Takahashi 1965; Tanji et al. 1978). Clearly, there is evolutionary pressure against too high a proportion of large axons, since they occupy relatively more brain volume and have higher metabolic demands. Perge et al. (2012) have suggested that where larger axons do exist, this may be to sustain a large number of different terminal arbors or to carry spike information at high frequency. Although there is evidence that fast PTNs arborize in a relatively focused manner onto spinal motoneurons (Buys et al. 1986; Fetz and Cheney 1980; McKiernan et al. 1998; Shinoda et al. 1976, 1981), we know nothing about the full extent of the arbor of fast vs. slow PTNs. Fast PTNs with large axons (say, those diameters >8 μm) can fire at higher instantaneous frequencies than slower PTNs (Evarts 1965), but their mean firing rates are still well below those predicted from the model presented by Perge et al. (2012).This model shows a linear relationship of mean firing rate to fiber diameter up to 3 μm but did not explore this for larger fibers, such as are found in the monkey CST.

An alternative explanation for the existence of these large fibers is the reduction of conduction delays (Perge et al. 2012; Tomasi et al. 2012). In primates, the largest fibers may be involved in control of actions such as skilled manipulation that are particularly sensitive to central delays (Racz et al. 2012; Venkadesan and Valero-Cuevas 2008). Large-bodied mammals such as cat, monkey, ape, and human have both very long CSTs and some large CST axons (>6 μm, up to 22 μm in humans; Kuypers 1981); such large fibers are completely lacking in smaller animals such as rats and mice (maximum diameter around 3 μm). It is known that corticospinal neurons with the thickest axons are located mostly in area 4 (van Crevel and Verhaart 1963), and this is also true of the callosal projection (Tomasi et al. 2012). It is interesting that in macaque M1, neurons (and by inference, their axons) projecting to the lumbosacral cord are significantly larger than those projecting to the cervical cord (Murray and Coulter 1981). It is also well established that Betz cells in the leg area of human motor cortex are larger than those in the arm area (Rivara et al. 2003; von Bonin 1949). The largest somata of macaque M1 corticospinal neurons, with diameters of 40–60 μm, are larger than those in postcentral areas 3b, 2, and 1 (up to 40 μm; Murray and Coulter 1981).

### The Enigma: What is the Function of the Many Slowest-Conducting PT fibers?

We are still left with an enigma: the functional properties of most PTNs remain elusive. They could be involved in one or more of a number of possible roles mediated by fibers of the CST (Lemon 2008), and there is evidence that slower PTNs in M1 are specifically involved in small-amplitude movements or position control (Fromm and Evarts 1981; Tanji et al. 1978). One distinctive feature of the macaque and human corticospinal system is the presence of direct cortico-motoneuronal (CM) projections; it is known that these originate from both fast and slow PTNs (Porter and Lemon 1993), and indeed, retrograde viral tracing shows that CM cells exhibit the same soma size range as all corticospinal neurons, with a distribution strongly skewed to small cell bodies (Rathelot and Strick 2006). Stimulation of the PT after a lesion partially interrupting the CST at mid-cervical levels evokes excitatory postsynaptic potentials (EPSPs) in hand motoneurons with latencies longer than those for normal monosynaptic CM responses. These EPSPs could be disynaptic and originate from C3–C4 propriospinal projections (Sasaki et al. 2004) but could also result from monosynaptic CM projections from slow uninjured corticospinal fibers (Lemon 2004; Maier et al. 1998). From a neurological standpoint, this is significant because of the large numbers of slow-conducting corticospinal fibers, particularly because fast fibers may be more susceptible to injury (Blight 1991; Quencer et al. 1992).

The cortical origin of neurons giving rise to fine axons in the PT is still unknown. Early work by Welch and Kennard (1944) established that all of the fibers in the PT have a cortical origin: a complete decortication of macaque monkeys resulted in complete degeneration of the ipsilateral PT. Häggqvist (1937) suggested that that many of finest PT axons probably originate outside the primary motor (M1) and premotor cortex: he showed that large numbers of fine PT axons (1–3 μm) survived lesions of areas 4 and 6 of the monkey cortex. Thus it could be that the lack of PTNs with conduction velocities below 5 m/s in M1, F5, and SMA (Figs. 4 and 5) is not only due to sampling bias in our recordings (cf. Humphrey and Corrie 1978) but also because relatively few of these corticospinal neurons are actually located in these areas. Support for this suggestion comes from neurophysiological studies in which it was possible to make recordings from cortical neurons with very slowly conducting axons (<5 m/s) but belonging to other classes of cortical projections, such as callosal neurons (Soteropoulos and Baker 2007; Waxman and Swadlow 1977). It could be that visceromotor projections from mesolimbic or cingulate areas of cortex to autonomic centers (Bacon and Smith 1993; Kennard, 1947; Levinthal and Strick 2012) are mediated by the slowest conducting axons.

Although only a tiny proportion of fibers in the macaque PT are unmyelinated, there are clearly a huge number of fine corticospinal fibers with diameters <1 μm (52% of the present sample). In 1978, Humphrey and Corrie emphasized that to understand completely the properties of the CST, special emphasis need to be put on studying the slowest conducting fibers, unseen by them in their study. The discussion above indicates that little has changed. Indeed, the present anatomical studies show that there are even more corticospinal fibers for which nothing is known of their physiology or their functions. If the whole population of fibers is seen as a continent, only the littoral has been explored, the hinterland is completely unknown.

### Conclusions

Our study emphasizes that the proportion of very thin corticospinal axons is even higher than estimated previously and that we still have little or no information about the activity patterns of the neurons giving rise to the thinnest PT axons. This will require specific studies to locate the cortical origin of these small neurons, using improved stimulation, recording, and other techniques to identify these small neurons. It may turn out that they can be found in large numbers in the nonmotor cortical areas that give rise to corticospinal projections. Once they have been identified, their functional contribution can be studied along lines similar to those successfully pursued for large corticospinal neurons.

## GRANTS

This work was funded by the Wellcome Trust, Volkswagen Stiftung, and UK National Centre for the Replacement, Refinement and Reduction of Animals in Research.

## DISCLOSURES

No conflicts of interest, financial or otherwise, are declared by the authors.

## AUTHOR CONTRIBUTIONS

L.F., P.F., M.A.M., A.K., P.A.K., K.N., R.L., and M.G. performed experiments; L.F., P.F., M.A.M., A.K., P.A.K., K.N., R.L., and M.G. analyzed data; L.F., P.F., M.A.M., A.K., P.A.K., R.L., and M.G. interpreted results of experiments; L.F., P.A.K., R.L., and M.G. drafted manuscript; L.F., M.A.M., A.K., P.A.K., K.N., R.L., and M.G. approved final version of manuscript; M.A.M., R.L., and M.G. conception and design of research; M.A.M., P.A.K., and M.G. prepared figures; M.A.M., A.K., P.A.K., R.L., and M.G. edited and revised manuscript.
